# Targeting neuronal MAPK14/p38α activity to modulate autophagy in the Alzheimer disease brain

**DOI:** 10.1080/15548627.2016.1238555

**Published:** 2016-10-07

**Authors:** John Alam, Wiep Scheper

**Affiliations:** a EIP Pharma LLC, Cambridge, MA, USA; b Department of Functional Genomics, Center for Neurogenomics and Cognitive Research, VU University, Amsterdam, The Netherlands; c Department of Clinical Genetics and Alzheimer Center, VU University Medical Center, Amsterdam, The Netherlands

**Keywords:** AKT, Alzheimer disease, APP/PS1 mouse, autophagy, BACE1, biomarkers, MAPK14, MTOR, p38α

## Abstract

Dysregulated autophagic-lysosomal degradation of proteins has been linked to the most common genetic defect in familial Alzheimer disease, and has been correlated with disease progression in both human disease and in animal models. Recently, it was demonstrated that the expression of MAPK14/p38α protein is upregulated in the brain of APP-PS1 transgenic Alzheimer mouse and further that genetic deficiency of *Mapk14* in the APP-PS1 mouse stimulates macroautophagy/autophagy, which then leads to reduced amyloid pathology via increasing autophagic-lysosomal degradation of BACE1. The findings resolve at least in the context of the APP-PS1 mouse, prior conflicting in vitro observations that have implicated MAPK14 in autophagic processes, and indicate that inhibition of MAPK14 enzyme activity has potential as a therapeutic approach to mitigate a critical physiological defect within neurons of the Alzheimer disease brain. Moreover, the findings suggest that biomarkers of BACE1 activity could be utilized to evaluate the effects of MAPK14 inhibition and other autophagy-inducing therapeutic approaches in human clinical studies, thereby potentially facilitating the clinical development of such agents.

The key hallmark of neurodegenerative diseases such as Alzheimer disease (AD) is the accumulation of aggregated, misfolded proteins. This accumulation is considered to be caused by both increased production of misfolded proteins, as well as impaired clearance of misfolded proteins and aggregates. Dysfunction of the autophagy-lysosomal system can be observed at early stages of pathology in the human AD brain^1^ and increases with disease progression.^2^ Interestingly, deficiency in PS1 (presenilin 1), mutations in which are the most common cause of familial AD, leads to lysosomal dysfunction.^3,4,5^ Modulation of the autophagy-lysosomal pathway is therefore an interesting target for the treatment of neurodegenerative disease,^6,7^ and proof of concept using genetic approaches has been obtained in different animal models. For example, in mouse models for AD amyloid pathology the phenotype can be rescued by increasing lysosomal degradation.^8,9^ As recently reviewed by Friedman and colleagues^6^ the most promising drug approaches involve both MTOR-dependent and MTOR-independent approaches, though the specific intervention points that would have the most disease impact remain to be defined. In addition, the autophagy-lysosomal system is an integral part of several important homeostatic stress pathways and therefore, for therapeutic applications, it is important to develop modulators of the pathway, rather than structural interventions in the core signaling.

A potential candidate for modulation of autophagy would be the α isoform of the stress-activated kinase MAPK14/p38α (mitogen-activated protein kinase 14), because it is activated in the AD brain^10^ and as discussed below has been implicated in autophagy regulation. However, the evidence to date on the role of MAPK14 in regulating autophagy is based on in vitro observations and, depending on the model and mode of intervention utilized, MAPK14 activation has been demonstrated to either inhibit or stimulate autophagy. Recently, Schnöder and colleagues^11^ employed neuron-specific genetic deletion of *Mapk14* in an APP (amyloid β [A4] precursor protein)-PS1 (presenillin 1) (APP-PS1) transgenic mouse model for AD and demonstrated increased autophagy and reduced amyloid pathology. This provides the first in vivo demonstration of the effects of selective reduction of MAPK14 activity on autophagy and suggests that therapeutic inhibition of MAPK14 has the potential to address the autophagic defect in Alzheimer disease.

The members of the p38 MAPK family (MAPK14/p38α, MAPK11/p38β, MAPK12/p38γ and MAPK13/p38δ) are activated in response to extracellular stimuli and, via intracellular transduction signaling networks and regulation of transcription/translation, play a pivotal role in many cell types in adapting to, and fine-tuning the response to, environmental stress.^12^ The MAPK14/p38α and MAPK13/p38β isoforms are most broadly expressed and their role is best defined as modulators of the innate immune system, particularly the promotion of pro-inflammatory cytokine production from macrophages; a context in which MAPK14 appears to be more critical than MAPK13. Rather than a direct effect on macrophage activation, MAPK14 appears to be involved in crosstalk between MAPK14 and the AKT-MTOR pathways downstream of the toll-like receptors; the net effect of which is a “tuning” of the AKT-MTOR pathway in response to environmental stimuli.^13,14,15^ An important implication is that MAPK14 does not determine the direction of the inflammatory response (i.e., proinflammatory vs. anti-inflammatory), which is determined by the AKT-MTOR pathway; rather MAPK14 determines the strength and duration of the response.^13-15^

One inherent limitation in defining a specific biological role of MAPK14 has been that *Mapk14* genetic knockout in mice is embryonic lethal due to an early defect in angiogenesis.^16^ This is caused by a defect in placental embryogenesis that results in poor delivery of nutrients to the embryo and is not due to defects in embryogenesis otherwise.^16^ In addition, most chemical inhibitors (e.g., “SB203850”) that have been utilized in laboratory experiments over the last 2 decades have poor selectivity for one or other of the isoforms, even when described as “selective MAPK14/p38α” inhibitors.^17^ Early observations with chemical inhibitors suggested that inhibition of MAPK14 would block autophagic flux in vitro, though subsequent studies clearly indicate that these observations are due to off-target effects as the evaluated inhibitors antagonize other kinases, whereas more selective MAPK14 inhibitors do not show a similar effect.^18^ Equally, studies that have evaluated the effects of depleting the *Mapk14* gene have indicated the effects of MAPK14 on autophagy appear to be context-specific; i.e., whether it stimulates or inhibits autophagy is dependent on the biological system and/or stimulus for autophagy. For example, *MAPK14* depletion using a siRNA approach identified MAPK14 as a negative regulator of both basal and starvation-induced autophagy in HEK293 cells via competing with ATG9 for binding to SUPT20/p38-interacting protein.^19^ In addition, MAPK14 activation inhibits autophagosome-lysosome fusion via phosphorylation of ATG5; and *MapK14*-deficient mouse embryonic fibroblasts demonstrate increased autophagic flux relative to wild-type mouse embryonic fibroblasts in response to starvation.^20^ Conversely, in the context of a very specific model of autophagy induction, glucose stimulation after starvation, MAPK14 activation appears to stimulate autophagy.^21^ Similarly, Wei et al. have shown that activation of the downstream MAPK14 targets MAPKAPK2/MK2 and MAPKAPK3/MK3 activates amino acid starvation-induced autophagy via BECN1 phosphorylation.^22^

In contrast to the periphery where it is expressed robustly in most cell types, in the adult healthy brain there is only low-level expression of MAPK14.^23^ Indeed, apparently as protection against maladaptive responses to neuronal stress, *MAPK14* transcription in adult neurons appears to be actively repressed by *Mir124* and *Mir128*.^23^ A significant advance in our understanding of the role of MAPK14 in the brain was made by Schnöder and colleagues, who specifically evaluated MAPK14 expression in neurons and the effects of *MAPK14*/*Mapk14* genetic deficiency in neuronal cells in vitro (SH-SY5Y cells) and in vivo (APP-PS1 transgenic mouse). As a first step, they confirmed previous observations that MAPK14 expression in neurons is low in wild-type mice, but significantly increased in the APP-PS1 mouse. One allele of *Mapk14* was deleted in the APP-PS1 mouse and one or both alleles of *MAPK14* in vitro, and in both contexts the reduction of MAPK14 activity decreases amyloid β levels. In addition, plaque pathology is reduced in the hemizygous *Mapk14*-deficient APP-PS1 mouse, compared to the APP-PS1 mouse with wild-type levels of MAPK14. Moreover, reduction of MAPK14 activity stimulates autophagy as both the ratio of LC3-II:LC3-I protein and the amount of BECN1 protein detected in the RIPA buffer-soluble brain homogenate are significantly higher in the APP-PS1 mouse that is heterozygous for *Mapk14*, compared to the APP-PS1 mouse with wild-type levels of MAPK14. Concomitantly, the BACE1/β-secretase 1 (β-site APP cleaving enzyme 1) protein levels and corresponding enzyme activity are significantly reduced. This reduction of BACE1 protein levels was demonstrated to be via MAPK14-mediated facilitation of lysosomal degradation of the BACE1 protein. Finally, the effects on lysosomal degradation of BACE1 appear dependent on stimulation of autophagy, as blocking autophagy by treating cells with 3-methyladenine or overexpressing dominant-negative ATG5 abolishes the effect of MAPK14 on BACE1 protein levels. However, unresolved in the article was the specific mechanism by which stimulation of autophagy leads to increased lysosomal degradation of BACE1.

The primary conclusions of the Schnöder article relate to the role of MAPK14 with regard to regulation of BACE1 protein levels and amyloid β generation. However, we think the implications with regard to the role of MAPK14 in stimulating autophagy in the context of the APP-PS1 mouse are equally interesting as they are the first in vivo demonstration of MAPK14 affecting autophagic processes in the brain, whereas the in vitro observations have been conflicting as to whether MAPK14 inhibition would stimulate or inhibit autophagy. Moreover, increased expression of BACE1 due to defective lysosomal proteolysis is only one consequence of a more fundamental failure of autophagy-lysosomal-mediated proteolysis in the APP-PS1 mouse.^24^ As such, the results suggest that targeting MAPK14 activity may be a means to ameliorate the defect in autophagic-lysosomal protein degradation that has otherwise been demonstrated in Alzheimer disease (Fig. 1).

**Figure 1.**
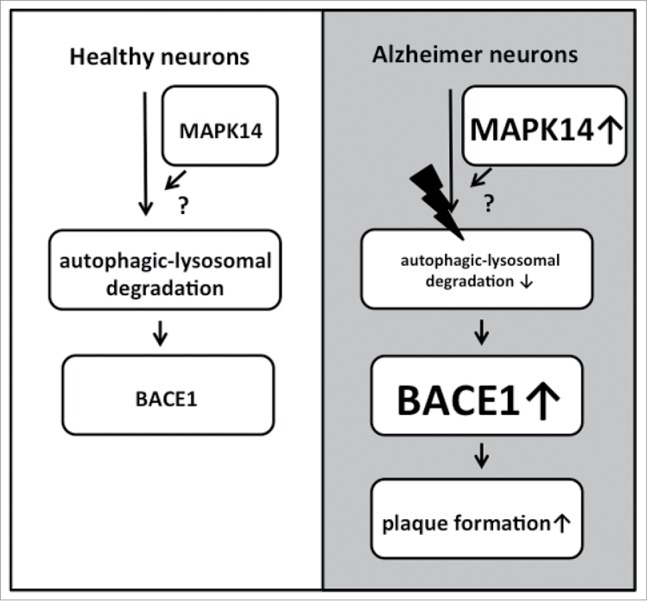
Hypothetical model for the involvement of MAPK14 in AD pathogenesis. The autophagic-lysosomal degradation of proteins is inhibited via modulation of a yet unidentified pathway, possibly involving AKT-MTOR. In healthy neurons, MAPK14 levels are relatively low, ensuring autophagic flux and degradation of BACE1. In AD neurons autophagic-lysosomal protein degradation is impaired (indicated by the lightning bolt). In addition, MAPK14 levels are increased, leading to further decreased autophagic flux, increased BACE1 levels and plaque formation. Reduction of MAPK14 suppresses the autophagic defect and thus reduces BACE1 levels and plaque formation. See text for further details.

Because regulation of autophagy is to a great extent dependent on the AKT-MTOR pathway,^25^ crosstalk between that pathway and MAPK14 is well established in other biological contexts (discussed above), one potential mechanism for the stimulation of autophagy resulting from MAPK14 deficiency is derepression of the AKT-MTOR pathway. Consistent with the effect of MAPK14 on autophagy being via modulation of another pathway is that while there are some effects on BACE1 protein with *Mapk14* deficiency in wild-type mice, the effects are more marked in the APP-transgenic mice. This leads us to suggest that the effect of reducing MAPK14 activity on autophagy is not necessarily direct stimulation of autophagy; rather, that it is reversing or modulating a pathway (e.g. AKT-MTOR) that inhibits autophagy in the pathological context of overproduction of amyloid β.

An additional finding of Schnöder is that BACE1 enzyme levels are regulated by the extent of autophagic-lysosomal degradation of the protein, which then affords opportunities for a human clinical trial biomarker to assess the effects of drugs that stimulate autophagy in the context of AD. As BACE1 enzyme inhibitors significantly decrease (by up to ~90%) cerebrospinal fluid concentrations of amyloid β peptides within 2 wk of treatment,^26^ a drug that stimulates autophagy in the context of Alzheimer disease, thereby reducing BACE1 enzyme levels, would potentially also relatively rapidly reduce cerebrospinal fluid amyloid β peptides levels. Furthermore, with protracted treatment, stimulation of autophagic-lysosomal degradation and subsequent reduction of BACE1 enzyme levels should replicate the APP-PS1 results and reduce amyloid pathology in the brain (which in the human context could be quantified with amyloid PET scanning).

MAPK14 as an Alzheimer therapeutic target has otherwise primarily been considered as an anti-inflammatory mechanism to target innate immune responses in the brain,^12,27^ particularly with regard to microglial activation; as well to reduce inflammation-induced synaptic toxicity.^28^ However, the neuroinflammatory response appears to function as a double-edged sword in neurodegenerative disease. This is also the case for MAPK14-mediated inflammatory responses: it has been argued that MAPK14-dependent microglial-mediated phagocytosis and clearance of amyloid plaques is protective in AD.^29^ In that regard, the findings of Schnöder suggest that the effects on BACE1 enzyme levels in the neuron due to inhibition of neuronal MAPK14 potentially would offset any deleterious effects on amyloid pathology resulting from inhibition of microglial MAPK14. The other major concern regarding therapeutically targeting MAPK14 has been the potential for toxicity. With non-blood-brain-barrier penetrant compounds the dose-limiting toxicity in clinical studies have been liver enzyme elevations and skin rash,^30^ which both can be avoided with compounds that minimize systemic drug exposure by preferentially distributing to the brain. An unspecified CNS toxicity has been reported in 1 of 2 animal toxicology species after more than 6 mo of administration of a CNS-penetrant MAPK14 chemical inhibitor.^30^ However, CNS toxicity has not been reported in clinical studies, probably because the toxicity in animals was reported to occur only at doses 10-fold higher than the highest doses utilized in the clinic.^30^ The lack of overt brain developmental defects resulting from neuronal deficiency of MAPK14 in the report of Schnöder provides additional assurance, but only human clinical studies will provide definitive answers for these toxicity concerns; as well the concerns regarding effects on microglial-mediated clearance of amyloid.

In conclusion, though not all the pieces of the puzzle have been put in place, the work of Schnöder and colleagues indicates that inhibition of MAPK14 has the potential to be a pharmacological approach to mitigate defects in the autophagy-lysosomal system, which has been proposed as a critical component of the pathogenesis of Alzheimer disease. The potential advantages of such an indirect approach to correcting disease-driven failure of autophagy are: (1) by not interfering directly in autophagy pathways the normal physiology of the autophagy-lysosomal system remains intact, and (2) it would be context- and/or disease-specific to settings where aberrant activation of MAPK14 contributes to the autophagy-lysosomal defect. As at least 1 brain-penetrant specific MAPK14 inhibitor is currently in imaging and cerebrospinal fluid amyloid biomarker-based phase 2a clinical trials in patients with AD (clinicaltrials.gov; NCT02423122 & NCT02423200), we may soon learn how these preclinical observations translate to human disease.

## Abbreviations

AD: Alzheimer disease
AKT/PKB: thymoma viral proto-oncogene
APP: amyloid β (A4) precursor protein
ATG5: autophagy-related 5
ATG9: autophagy-related 9
BACE1: β-site APP cleaving enzyme 1
MAPK: mitogen-activated protein kinase
MTOR: mechanistic target of rapamycin (serine/threonine kinase)
MAPK14/p38α: mitogen-activated protein kinase 14
PS1: presenilin 1
